# Supplementary material for: Dynamic transcriptomic profiles of zebrafish gills in response to zinc supplementation
Source: BMC Genomics. 2010 Oct 11;11:553. doi: 10.1186/1471-2164-11-553 (PMC3091702; doi:10.1186/1471-2164-11-553)
Supplement: Additional file 2 — Interactive Direct Interaction Network representing the molecular interactions between zinc, copper, iron, calcium and proteins encoded by transcripts changed by zinc supplementation. Mini web-site containing index.html and hyperlinked pages in subdirectory describing a Direct Interaction Network automatically generated based on curated interactions contained within the proprietary PathwayArchitect database. Ovals represent proteins and the circles symbolize metal ions. Objects are coloured by their abundance in zebrafish at the time-point they were significantly different from the control is a scale from -4 fold (dark green) to +4 fold (dark red). Where significant differences were found at more than one time-point, the colour overlay shows expression at the first instance. Dark blue squares denote 'binding', and light blue squares 'expression'; green squares stand for 'regulation', green diamonds for 'metabolism', and green circles for 'promoter binding'. Arrow heads indicate directionality of the interaction where annotated. All nodes and edges can be further interrogated by selecting the relative area of the image. [file 1471-2164-11-553-S2.zip › PathwayArchitect Zn xs DIN/693695.html]

# REGULATION:

|  |  |
| --- | --- |
| Type | REGULATION |
| Effect | Positive |


---

|  |  |
| --- | --- |
| Score | 0 |


---

|  |  |
| --- | --- |
| Reference Count | 6 |


---

|  |  |
| --- | --- |
| Mechanism | Unknown |


---

|  |  |
| --- | --- |
| Reference:0 || Sentence | "To understand the molecular mechanisms responsible for TGF-beta1 response on collagenase-3 promoter activity, a functional analysis of the promoter region of the collagenase-3 gene was carried out, and we identified the distal runt domain (RD) and proximal RD/activator protein-1 (AP-1) sites as necessary for full TGF-beta1-stimulated collagenase-3 promoter activity." |
| PMID | 15084595 |
| Year | 2004 |
| Species | Human |
|  | Rat |
| Journal | J Biol Chem |
| RefScore | 1 |
| Source | PArchNLP |
  |
|  |  |


---

|  |  |
| --- | --- |
 Reference:1 || Sentence | "MMP-13 mRNA up-regulation was preceded by up-regulation of c-jun and c-fos mRNA." |
| PMID | 11751895 |
| Year | 2001 |
| Species | Rat |
| Journal | J Biol Chem |
| RefScore | 1 |
| Source | PArchNLP |
  ||  |  |


---

|  |  |
| --- | --- |
 Reference:2 || Sentence | "We demonstrate that this differential effect of TGF-beta on collagenase gene expression is due to a cell type-specific induction of distinct oncogenes of the Jun family, which participate in the formation of AP-1 complexes with different trans-activating properties." |
| PMID | 8631909 |
| Year | 1996 |
| Species | Mouse |
|  | Human |
| Journal | J Biol Chem |
| RefScore | 0 |
| Source | PArchNLP |
  ||  |  |


---

|  |  |
| --- | --- |
 Reference:3 || Sentence | "Mutation of either the activator protein-1 or the runt domain binding site resulted in the inability of c-Fos and c-Jun or core-binding factor a1 to increase collagenase-3 promoter activity, suggesting that there is cooperative interaction between the sites and the proteins." |
| PMID | 10779518 |
| Year | 2000 |
| Species | Rat |
| Journal | J Biol Chem |
| RefScore | 0 |
| Source | PArchNLP |
  ||  |  |


---

|  |  |
| --- | --- |
 Reference:4 || Sentence | "Finally, by using electrophoretic mobility shift assays and antibody supershift analysis, we propose that c-Fos, c-Jun, and JunD may play major roles in the collagenase-3 activation by TGF-beta1 in human fibroblasts." |
| PMID | 9545314 |
| Year | 1998 |
| Species | Human |
| Journal | J Biol Chem |
| RefScore | 1 |
| Source | PArchNLP |
  ||  |  |


---

|  |  |
| --- | --- |
 Reference:5 || Sentence | To understand the molecular mechanisms responsible for TGF-beta1 response on collagenase-3 promoter activity, a functional analysis of the promoter region of the collagenase-3 gene was carried out, and we identified the distal runt domain (RD) and proximal RD/activator protein-1 (AP-1) sites as necessary for full TGF-beta1-stimulated collagenase-3 promoter activity. |
| Year | 2004 |
| PMID | 15084595 |
| Species | Human |
|  | Rat |
| Journal | J Biol Chem |
| RefScore | 2 |
| Source | PArchNLP |
  |


---

|  |  |
| --- | --- |
